# When higher doses in opioid replacement treatment are still inadequate – association to multidimensional illness severity: a cohort study

**DOI:** 10.1186/1747-597X-9-13

**Published:** 2014-02-28

**Authors:** Jens Reimer, Eduard Boniakowski, Christian Bachner, Bernd Weber, Wieland Tietje, Uwe Verthein, Stephan Walcher

**Affiliations:** 1 Centre for Interdisciplinary Addiction Research, University of Hamburg, Martinistrasse 52, 20246 Hamburg, Germany; 2 Practice for General Medicine and Addiction Medicine, Königswiesenweg 21, 93059 Regensburg, Germany; 3 Practice Centre Friedrichsplatz, Friedrichsplatz 2-3, 34117 Kassel, Germany; 4 Practice for General Medicine, Stockholmer Strasse 52, 28719 Bremen, Germany; 5 Practice for Addiction Medicine, Kaiserstrasse 1, 80801 Munich, Germany

**Keywords:** Opiate dependence, Substitution treatment, Dose adequacy, Illness severity, Sexual functioning

## Abstract

**Background:**

Opioid replacement treatment (ORT) with methadone is regarded as gold standard in the treatment of opioid addiction. Treatment doses of 60 mg methadone per day and above are associated with better treatment retention and reduction in the use of heroin and cocaine. However, an absolute dose level cannot function as parameter for adequate dosing. This study aims to determine dose adequacy in a sample of patients on stable methadone treatment, and to relate dose adequacy to disease severity.

**Methods:**

This study was designed as open prospective cohort study over 12 months, with baseline data reported here. Patients on stable substitution treatment with methadone (Eptadone®) were consecutively included. Medical and socio-demographic data were gathered and the instruments Opiate Dosage Adequacy Scale (ODAS), European Addiction Severity Index (EuropASI) and the Derogatis Interview for Sexual Functioning – Self Report (DISF-SR) were applied.

**Results:**

Five hundred and sixteen subjects, who received on average 60.3 (±30.4) mg methadone per day, were included. According to ODAS, 40.6% suffered from an inadequate dosing, and 59.4% had an adequate dose. Patients with an adequate dose received on average 57.8 (±27.5) mg methadone per day, whilst patients with an inadequate dose received on average 70.6 (±33.0) mg per day. The frequencies of patients with methadone doses of less than 60 mg per were 45.4% in the inadequate and 60.6% in the adequate group. The inadequate group suffered from a statistically significant higher burden of addiction related problems in all EuropASI domains. Sexual functioning did not differ by adequacy group, but women suffered from more pronounced sexual dysfunction as compared to men.

**Conclusion:**

A high frequency of inadequate dosing was found in this sample of patients on ORT. Higher disease severity should alert for possible need of even higher methadone doses. The tendency to low methadone doses warrants further research in the treatment system. Higher methadone doses are not related to increased sexual dysfunction. Sexual dysfunction, especially in women, should be considered in treatment.

## Background

Dependency from illegal opioids (i.e. heroin) constitutes a severe and chronic disease, which is associated with high mortality and co-morbidity, as well as severe functional impairment in various life dimensions [1-3]. Illegal opioid use increases mortality in young adults up to 15 times [4]. Co-morbidity in heroin users comprises mental illnesses such as depression, anxiety, and personality disorders, as well as somatic states such as hepatitis C virus (HCV) and human immunodeficiency virus (HIV) infections, abscesses, pulmonary and cardiovascular problems. Impairment of functioning goes along with high unemployment rates, instable partnership and housing, criminal activities and legal problems [5].

Opioid replacement treatment (ORT) includes the substitution of the illegal opioid by a prescribed opioid, favorably with good μ-receptor activity and longer half-life to avoid euphoric and withdrawal symptoms. ORT reduces mortality by a factor three to eight compared with untreated heroin addicts, and is also associated with reductions in illicit drug use, the frequency of injecting, HIV-transmission and criminal activity [1,6,7]. Both methadone and buprenorphine are effective medications in ORT; whilst methadone seems to have some advantages in terms of treatment retention and reduction of heroin and cocaine consumption in case doses are in the range of 60 to 100 mg per day [8-10]. However, an absolute dose level cannot serve as an indicator of treatment sufficiency, and should additionally be correlated to clinical and patient reported parameters. In this context, Trujols and colleagues suggested to distinguish between the concepts *holding dose*, *dose adequacy*, *satisfaction with methadone as a medication*, and *satisfaction with treatment*[11,12]*.* The two latter dimensions represent subjective phenomena, while the first two constructs include subjective and objective phenomena, with *dose adequacy* as a construct that can be measured by a validated instrument [13].

The application of such an instrument may pave way for a patient centered approach, also in substitution treatment. Due to the nature of addiction with loss of control over substance use, treatment approaches were and still continue to be of paternalistic nature to provide structure to the patient and help to control the disease. Nevertheless, patient-centered approaches have also entered substitution treatment, and were associated with increased treatment satisfaction [12]. As a sole reliance on patient requests would oftentimes interfere with treatment aims, the combined subjective and objective assessment of dose adequacy could serve as a valid basis to enable patient participation.

The influence of genetic variability on required methadone dose is well known [14-16], but may not explain all of the variance [13]. Besides genetic and neurochemical factors, multidimensional psychosocial disease severity may have an effect on required dosing. In the severely ill and socially disintegrated sample of the German study on heroin-assisted treatment, the average dose in the methadone control group was 100 mg per day, a rather high dose for the German treatment system [5,17].

This study aims to determine the frequency of adequate or inadequate methadone dose and possible clinical as well as psychosocial and legal correlates as a measure of disease severity in routine methadone replacement treatment. In this context, also sexual dysfunctioning as a common, but neglected condition in this patient group, which has been connected to methadone dose, was assessed as a relevant criterion of physical and mental health [18-21].

## Methods

The study followed a prospective observational cohort design in 14 German substitution practices. Experienced practices within the research network of the Centre for Interdisciplinary Addiction Research of Hamburg University and outside the heroin-assisted treatment program were asked for participation, site selection was based on expression of intent and potential numbers of eligible patients. Subjects diagnosed with opioid addiction (F11.2) according to the International Classification of Diseases-10 [22] and substituted with methadone (Eptadone®) for at least one month prior to study entry were consecutively included. Additional inclusion criteria comprised age of 18 years or older and provision of written informed consent. Patients were excluded in case of incapability to meet requirements of the study protocol (attend visits, understanding of questionnaires), decompensated mental disorders, non-drug related seizures and acute or severe somatic disorders such as acquired immunodeficiency syndrome, tuberculosis, acute hepatitis, sepsis. The study duration was 12 months. Study visits were scheduled for baseline (t_0_), month 3 (t_1_), month 6 (t_2_), month 12 (t_3_). The baseline visit comprised socio-demographic data, medical anamnesis and examination, medication, routine laboratory parameters, drug tests and application of the diagnostic instruments European Addiction Severity Index (EuropASI), Opiate Dosage Adequacy Scale (ODAS), and Derogatis Interview for Sexual Functioning-Self Report (DISF-SR). The diagnostic instruments were applied and basic medical parameters were gained at t_0_, t_1_, t_2_, and t_3_. Diagnostic instruments were handed out by nurse specialists, patients filled out the instruments during their waiting time in the practice in a secluded room. In this article, only baseline data are reported.

The EuropASI is a validated and reliable instrument to measure drug-related consequences in social, health and legal dimensions, i.e. somatic health, work and economic situation, alcohol use, substance use, legal situation, family and relationships, and mental health [23,24]. The ODAS scale represents a validated instrument to measure the dose adequacy of opiate medication in substitution treatment [25]. It recently has been adapted to the German background [13]. ODAS consists of six dimensions and covers the areas heroin consumption, narcotic blockade, opiate withdrawal syndrome mental and somatic, craving for heroin, and methadone overdosing. The dimensions include items to be handled by the physician based on the patient anamnesis and items to be answered by the patient. Scores range between 1 (worst) and 5 (best). The dose is regarded as adequate, in case scores of 4 or 5 are yielded in each item. The DISF-SR is a validated and reliable 25-item self-report instrument, which measures sexual functioning in five dimensions, i.e. sexual cognition/fantasy, sexual arousal, sexual behavior/experience, orgasm, and sexual drive/relationship [26].

The study was conducted in accordance with the declaration of Helsinki and its subsequent revisions including those of Seoul 2008. It was approved by the ethics committee of the Physician Chamber Hamburg, Germany and additionally all physician chambers ethics committees responsible for study sites outside the state of Hamburg. Patients provided an informed written consent and were instructed that they may terminate study participation at any time without providing reasons and without negative consequences for their treatment. Patients were included into the study between October 2010 and January 2012. Within this paper, only baseline (t_0_) data were analysed.

Data analysis was performed with the SPSS 20 statistical package. Data are presented in a descriptive way, statistical analyses were performed to stratify by dose adequacy and in case of sexual functioning by gender. The Mann–Whitney test, student t-test, Chi^2^- or Fishers exact test were used for analysis of statistical significance. The level of significance was set at p < .05.

## Results

Five hundred and sixteen subjects with an average age of 36.7 (±30.4) years were included in the study (Tables 1 and 2). Overall, the methadone dose according to the ODAS scale can be regarded as adequate; the scores for opiate dose adequacy were 4.6 (±0.7) for heroin consumption, 4.5 (±0.8) for narcotic blockade, 4.2 (±1.2) for physical opiate withdrawal, 4.3 (±1.2) for mental opiate withdrawal, 5.0 (±0.2) for craving for heroin, and 4.7 (±0.8) for methadone overdose. However, for heroin consumption, 7.2% failed to reach the satisfactory scores of 4 or 5, for narcotic blockade this frequency was 11.2%, for physical opiate withdrawal 25.3%, for mental withdrawal 21.1%, for craving for heroin 17.0%, and for methadone overdose 8.9%, resulting in an overall frequency of inadequate dosing of 40.6%.

**Table 1:** Sociodemographic data (n = 516)

	
Average age, years (SD)	36.7 (8.7)
Male gender, %	74.2
Ethnicity, %	
Caucasian	97.2
African	0.6
Asian	2.2
Marital status, %	
Single	75.8
Married	12.0
Divorced	12.2
Living situation, %	
Alone	69.0
With family	24.6
Homeless	0.6
Other	5.7
Education, %	
8 - 9 years school	64.2
10 years school	22.8
13 years school	4.7
University degree	1.8
Other	6.5
Vocational situation, %	
Unemployed	59.1
Employed	34.2
Other (student, homemaker)	6.7

**Table 2:** Substance use and psychotropic medication

	
Average daily methadone dose, mg (SD)	63.0 (30.4)
Urinalysis (n = 420), positive, %	
Heroin and other illicit opioids	36.4
Methadone	98.6
Cocaine	4.3
Benzodiazepines	25.0
Cannabinoids	30.0
Psychotropic medication (n = 483), yes, %	17.0

**Figure 1:**
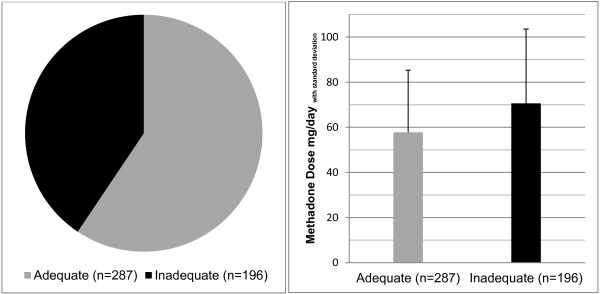
Dose adequacy (ODAS) and absolute methadone dose.

**Figure 2:**
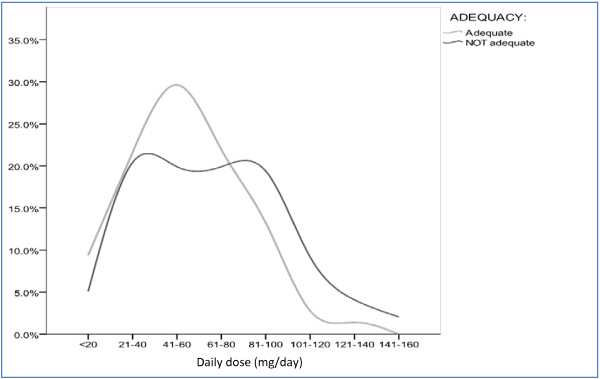
Methadone dose distribution by dose adequacy.

The scores of the adequate and inadequate dosing group differed significantly in the items consumption of heroin (4.7 ± 0.5 vs. 4.5 ± 0.8; t (481) = 3.514; p < .001), narcotic blockade (4.7 ± 0.5 vs. 4.3 ± 1.0; t (481) = 4.805; p < .001), physical opiate withdrawal (4.9 ± 0.3 vs. 3.2 ± 1.4; t (481) = 17.117; p < .001), mental opiate withdrawal (4.9 ± 0.4 vs. 3.5 ± 1.4; t (481) = 13.714; p < .001), craving for heroin (4.6 ± 0.5 vs. 3.9 ± 1.2; t (481) = 7.517; p < .001), and methadone overdose (5.0 ± 0.2 vs. 4.4 ± 1.2; t (481) = 6.869; p < .001). Also the sum score for all items differed significantly between groups, it was on average 28.7 (±1.6) in the adequate group and 23.7 (±3.1) in the inadequate group (t (481) = 20.755; p < .001). Absolute methadone dose was on average 57.8 (±27.5) mg per day in the adequate group and 70.6 (±33.0) mg per day in the inadequate group (Figure 1). In the adequate group, 39.4% received doses of 60 mg daily and above, in the inadequate group, this frequency was 54.6%. In general, the dose curve of the inadequate group showed a right shift, with two subtle peaks, one in the lower dose area around 40 mg/day and one in the higher dose area around 100 mg/day, whereas the adequate group followed a reversed u-form with a peak around 60 mg/day, that phases out with a right shoulder (Figure 2).

**Figure 3:**
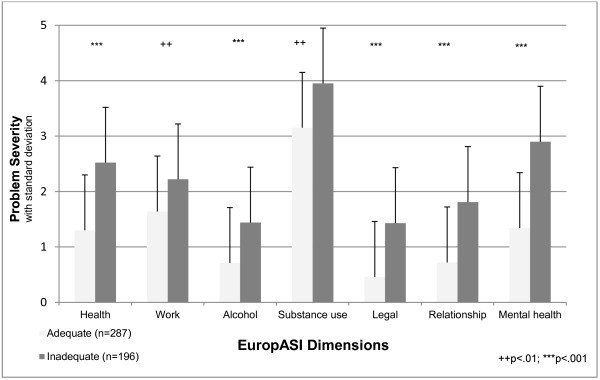
EuropASI scores by dose adequacy; health: t (481) = -5.34; p < .001, work: t (481) = -2.96; p = .003, alcohol: t (481) = -3.80; p < .001, substance use: t (481) = -3.53; p = .001, legal: t (481) = -5.34; p < .001, relationship: t (481) = -5.68; p < .001, mental health: t (481) = -6.50; p < .001.

Patients with inadequate doses suffered from a higher burden of addiction related problems as indicated by the EuropASI (Figure 3). Moreover, in four out of seven dimensions (alcohol problems, legal problems, relationship problems, and mental health problems), the score differed by factor two in disadvantage for the inadequate group. In the area legal problems, the score differed even by the factor three in disadvantage for the inadequate group, and items with a distinct difference included being on probation (24.5% vs. 7.2%; chi^2^ (1, N = 475) = 28.16; p < .001), awaiting trial or judgment (17.9% vs. 4.3%; chi^2^ (1, N = 475) = 23.73; p < .001), and number of days through the last month in illegal activities (2.0 ± 6.2 vs. 0.4 ± 2.9; t (481) = 3.31; p = .001). Mental symptoms with a statistically significant higher frequency in the inadequate group covered days with mental problems within the last month (6.6 ± 11.1 vs. 2.4 ± 6.6; t (481) = 4.78; p < .001), severe anxiety or agitation (33.2% vs. 20.9%; chi^2^ (1, N = 474) = 9.05; p = .003), concentration problems (37.8% vs. 17.6%; chi^2^ (1, N = 474) = 24.24; p < .001), difficulties in controlling violent behavior (8.7% vs. 3.6%; chi^2^ (1, N = 474) = 5.52; p = .019), need for drug prescription for psychological problems (25.0% vs. 15.8%; chi^2^ (1, N = 474) = 6.13; p = .013), depression and isolation (26.0% vs. 15.5%; chi^2^ (1, N = 474) = 8.05; p = .005), paranoid ideation (5.6% vs. 0.7%, chi^2^ (1, N = 474) = 10.32; p = .001), memory problems (17.9% vs. 6.1%; chi^2^ (1, N = 474) = 16.23; p < .001), and suicidal thoughts (3.1% vs. .4%; chi^2^ (1, N = 474) = 5.77; p = .016).

**Figure 4:**
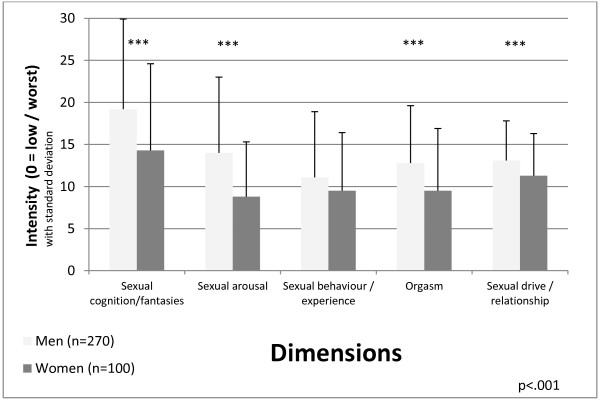
Sexual functioning (DISF-SR) by gender; cognition/fantasies: t (368) = 3.96; p < .001, arousal: t (368) = 6.19; p < .001, behaviour/experience: t (368) = 1.90; p = .059, orgasm: t (368) = 3.94; p < .001, drive/relationship: t (368) = 3.30; p = .001.

Concerning sexual functioning as measured with the Derogatis self-report interview, there were hardly any statistically significant differences between the adequate and the inadequate group in both gender groups, except for orgasm, which was better in men with inadequate dose (14.3 ± 6.5 vs. 11.8 ± 6.7; t (268) = 3.03 p = .003). Despite higher methadone doses, sexual functioning in the inadequate dose group did not differ from the adequate group with lower methadone doses. In detail, the inadequate group even reached non-significantly better scores in all dimensions (sexual cognition/fantasy, sexual arousal, sexual behavior/experience, orgasm, sexual drive/relationship) in men and in four out of five dimensions (sexual cognition/fantasies, sexual arousal, sexual behavior/experience, sexual drive/relationship) in women. On the other hand, there was a gender effect, and women scored statistically significantly worse compared to men in four out of five dimensions (Figure 4) and the sum score (sum score women vs. men 53.3 ± 28.8, 70.2 ± 31.6; t (368) = 4.90; p < .001).

## Discussion

This study examined *dose adequacy*, addiction severity and sexual functioning in a larger sample of opioid dependent patients on stable substitution treatment with methadone (Eptadone®). The study was conducted in 14 experienced addiction centres in Germany. According to the ODAS scale, two fifths of the patients received an inadequate dose. To meet the aim of ORT, namely treatment retention and reduction of heroin and cocaine consumption, doses of at least 60 mg methadone per day are required [8-10]. On average, the inadequate group received higher doses as compared to the adequate group (70.6 mg vs. 57.8 mg). However, doses of 60 mg methadone per day and above were received by only 39.4% in the adequate and 54.6% in the inadequate group. Additionally, in the inadequate group, the dose curve displayed one peak around 40 mg/day. The low percentage of patients with doses above the efficacy minimum of 60 mg methadone per day comes somewhat as a surprise, as there are no financial or legal constraints on methadone dosing. On the other hand, the ORT guidance of the German National Physician Chamber still ranks abstinence besides harm reduction and health improvement among the priority aims [27]. The combination of inherent abstinence orientation with treatment overregulation and paternalistic treatment delivery as well as possible motives on the patients’ side (desire for additional heroin use, negative aspects of methadone maintenance) might contribute to (too) low methadone doses [28,29]. These factors could add to low methadone doses around 40 mg/day especially in ‘problematic’ patients with pronounced health and legal problems as found in the inadequate group.

From the clinical view, it is important to note, that patients in the inadequate group received higher methadone doses. It can be assumed that physicians and their treatment team perceived the need and administered higher doses. However, the inadequate group still suffered especially from physical and mental opiate withdrawal, which indicates the need for even higher doses. At this point, in the context of above mentioned regulations in Germany, physicians might be reluctant to further adapt the dose to patients’ needs. The inadequate dose group scored worse in all EuropASI domains, which points at a higher disease severity and social disintegration. Higher frequency of mental symptoms in the inadequate group may be a sign of the use of methadone as a psychotropic medication not only for treatment of opiate dependence, but also for relief of further mental pain. Moreover, higher doses of methadone in combination with increased alcohol consumption might also be used to blind out legal problems. Poor general health status should alert for the need to retain the patient in maintenance treatment also by means of adequate dosing to further improve the patient’s health status. Alike, socially disintegrated and severely co-morbid methadone patients in the German heroin trial needed increased methadone doses [5,17]. In case of patient stabilization, vocational rehabilitation should be focused. Better co-operation between substitution physicians, the health care and the psychosocial support system is needed.

Sexual functioning did not differ between the inadequate and the adequate group, except for one dimension (orgasm) in men in favor for the inadequate group. Despite wide lack of a statistically significant difference, it is noteworthy that the inadequate group with higher methadone doses scored better than the adequate group in all five domains in men and in four dimensions in women. The relationship between methadone dose and severity of sexual dysfunction remains controversial, some studies found a relationship [20], whilst others did not [18,21]. For the clinician it is important to know, that sexual dysfunction is common in methadone substitution, but does not follow a strict dose-symptom severity relationship. It seems that doses of 100 mg per day and above could put especially male patients at an additional risk for development of sexual dysfunction [18]. For the first time, this study explored sexual functioning in a larger sample of female methadone substituted subjects. Compared to men, women had worse sexual functioning, and areas with marked impairment in women comprise ‘sexual cognition/fantasies’ and ‘sexual arousal’, which points at a combination of decreased cognitive and physiological functioning. On the background of high frequencies of childhood sexual abuse and intimate partnership violence in opiate dependent women, aforementioned symptoms may be regarded as defense mechanisms [30]. Further, a differential pharmacologic effects of methadone on sexual functioning in men and women warrant attention as well as frequencies of sexual abuse in opioid dependent men. ORT should take trauma-specific aspects into account [30].

It should be noted that the inclusion criteria only required a stable treatment with methadone of at least one month prior to study entry, so the study group may not necessarily represent long-term stable opioid substituted patients. Longitudinal data, not presented here yet, may allow for a more thorough evaluation.

## Conclusion

The introduction of the concept of *dose adequacy* provides additional relevant information in maintenance treatment. Even in case the clinical impression leads to administration of higher doses, these might not be adequate in terms of narcotic blockade, suppression of withdrawal symptoms, craving, and overdose. High disease severity in mental, physical, social and legal domains should alert for possible need for higher substitution medication doses.

## Competing interests

Author JR received speakers or consultant honoraria or research funding from Molteni Farmaceutici, Reckitt-Benckiser and Sanofi-Aventis. Authors EB and CB received compensation from Molteni Farmeceutici for study expenses. Author BW received speaker’s or consultant honoraria or study compensations from Hexal, Molteni Farmaceutici, Reckitt Benckiser, Sanofi-Aventis. Author WT received study compensations from Molteni Farmaceutici and Reckitt Benckiser. Author UV declares no conflict of interest. Author SW received speakers or consultant honoraria and study compensations from Molteni Farmaceutici and Reckitt Benckiser. Farmaceutici financially supported the preparation of this article, including the article processing charge.

## Authors’ contributions

Authors JR and SW designed the study, JR wrote a first draft of the article. Authors EB, CB, BW and WT collected data, revised and gave final approval for the manuscript. UV reviewed and approved the manuscript. All authors read and approved the final manuscript.
